# Supplementary material for: Widely Targeted Metabolomics and Network Pharmacology Reveal the Nutritional Potential of Yellowhorn (Xanthoceras sorbifolium Bunge) Leaves and Flowers
Source: Foods. 2024 Apr 21;13(8):1274. doi: 10.3390/foods13081274 (PMC11049039; doi:10.3390/foods13081274)
Supplement: Supplementary file 1 [file foods-13-01274-s001.zip › Supplementary materials.pdf]

Table S3 Gene symbol of target proteins interacts with non-volatile differential metabolites in flowers and leaves by network pharmacological analysis.

	Gene symbol
Flower	NFKB1, APEX1, HIF1A, PSMB1, KLF5, GPR55, KDM1A, NR1I2, CNR1, NFE2L2, TLR4, NTRK3, PTGS1, TOP2A, CLK4, CHUK, SCN2A, SLC6A5, SCN3A, NR3C2, ADAM10, CDK1, PDGFRA, CTSD, DPP9, FPR2, CACNA1B, PIK3R1, DUSP3, C5AR1, RORB, TFPI, ADORA1, GRIN1, TRIM24, PRKCD, GRIA2, METAP2, CYP3A4, CFTR, AURKB, SCD, CSNK2B, PDE3A, CNR2, HSP90AA1, GRK5, RXFP1, NTSR2, PSMB9, ACHE, CACNA1H, WDR5, S1PR5, PLAU, CCR1, CCR2, CYSLTR2, ITK, AOC3, FPRL2, FPR1, SAE1, PRMT5, RPS6KA3, NR2E3, GPR6, MAP2K2, QRFPR, SCN4A, F13A1, AR, HDAC5, PRCP, PRSS1, AXL, CDC25C, GLRA1, CCNE1, KEAP1, PRKAA1, ITGB1, GRB2, CASP8, CAPN1, GPR17, MC4R, ACACA, CACNA1C, TNK1, NOTUM, TDP1, HSD17B10, ALOX12, THRA, TTR, MAOA, FFAR4, ANPEP, LDHB, GPBAR1, HPGD, HDAC2, BLM, PIN1, GUSB, KCNA5, MDM4, PDE11A, KDM4C, PTPN7, ACVRL1, AVPR1B, SLC2A1, CAMKK2, PDGFRB, KIF11, PRKCZ, DHODH, CBX4, CHKA, GLO1, ENPP1, KDM4A, SERPINE1, PLAT, FAAH, PDE3B, P2RX4, S1PR4, GLS, NOX1, TYRO3, PTPN11, HTR2C, NPC1, NAAA, DCUN1D1, PTGER1, NR4A1, MTOR, MIF, CREBBP, ESR2, HDAC8, DYRK1A, RPS6KA1, NLK, CXCR4, ZAP70, AKR1C1, ERN1, STAT1, MAPKAPK2, DRD1, GBA2, TBK1, CDK2, SLC1A3, ACVR1, DPP7, CDK5, LGALS3, CHRM2, RPS6KA6, ADRB1, FCGRT, MME, SLC1A1, CHEK1, ERAP1, HSP90AB1, ACE, AAK1, GCK, NQO2, MMP12, NOS2, PTGER2, CHRM1, ABCC1, SLC1A2, HDAC10, EGLN1, CYP19A1, MCHR1, CDC25B, TACR1, GSK3A, GABRA1, DNMT1, PPIA, XDH, ACVR1B, MELK, TNIK, EIF2AK1, CLK2, ALOX5, MC5R, YES1, BRDT, ACACB, AKR1B1, GBA, IMPDH2, HRH3, CHRM3, TBXA2R, PTPN1, PTPN2, ATG4B, TLR8, HDAC7, TACR2, SLC40A1, SLC9A1, AKR1C3, OPRK1, CHRM4, MAP3K14, STK3, ADK, PNMT, CHRNB4, CHRM5, PRKACA, KLK1, RAB9A, NT5E, TYMS, CLK1, PTGS2, HDAC11, TOP1, KDM5A, MINK1, HDAC9, SIRT1, PKN2, CHRNB2, FASN, ADORA2B, BMPR1A, PKN1, PARP2, CSNK1E, HDAC3, ULK3, TMPRSS6, GSTP1, NEK2, HSD11B1, CDK4, KLK7, ABCG2, NOS3, ULK1, KIT, PSMB2, PDE9A, HPRT1, APLNR, IL23R, SLC6A2, MAP4K4, HRH4, HSD11B2, CSNK2A2, MARK4, GPR35, PHF8, KDM5C, TYMP, TBXAS1, CHRNA7, RPS6KA5, PTGES, CA3, CDK7, TDO2, TRPM8, CHEK2, AKT3, MAP3K11, HDAC4, KLK5, CSNK1D, DYRK1B, KLKB1, FDPS, BRD2, PKMYT1, GRM2, MAOB, FYN, DUT, TAOK1, LNPEP, GHSR, STING1, CYP2A6, P2RX7, CTSS, LYN, ALK, HTR1A, FGR, DPP8, DAO, GSK3B, ADAM17, PYGL, CHRNA1, IRAK1, AKR1C2, CASP6, EPHB2, EPHB3, CETP, DOT1L, MAPK1, OPRD1, CCR5, OPRM1, PIK3CD, P2RY12, S1PR3, ARG2, SPHK1, BTK, AKT2, PIK3CB, FAP, ABL1, MAP3K5, SLC5A1, APH1A, PARP1, PLA2G2A, PRKCA, FBP1, TGFBR2, F12, PAK4, APP, PDE4D, DPP4, ATR, NR1H4, NR3C1, CDC7, DCK, SLC6A4, STAT3, P2RY4, NOX4, FNTA, PRMT1, PDE5A, F2R, CFD, TGM2, PPARD, VDR, SIRT3, FLT1, CXCR6, PTK2B, SPHK2, DRD2, BMP2K, TYK2, RET, GAK, EZH2, HTR3A, KDM6B, PREP, KCNK9, and NAMPT.
Leaf	BLM, GPR55, TRIM24, PTGS1, NFKB1, CTSD, KDM1A, STAT3, APEX1, CYP3A4, NR3C2, PTPN2, CLK4, ADAM10, CDK5, HSD17B10, GLRA1, CSNK2B, HSP90AB1, NTRK3, FPR2, TLR4, NTSR2, PDE3A, CDC25B, SCN3A, PIK3R1, SCN2A, KLF5, TOP2A, GRIN1, AKR1C3, CNR2, AR, CLK1, PDGFRA, SLC9A1, FPR1, ITK, CACNA1B, TACR2, FPRL2, DPP9, GPR6, NR4A1, ADORA1, PROC, PRCP, CPT2, CYSLTR2, S1PR5, TERT, CHRM4, PLAT, CACNA1H, HDAC7, TLR8, QRFPR, RPS6KA1, CDC25C, AURKB, TFPI, MTOR, C5AR1, SAE1, PTK2B, CHRM1, PTGER1, PSMB9, STING1, DPP8, MARK4, SPHK1, CHRM5, SLC6A5, THRA.

Table S3 Gene symbol of target proteins interacts with non-volatile differential metabolites in flowers and leaves by network pharmacological analysis (continued).

	Targets
Leaf	ATG4B, CDK1, WDR5, AVPR1B, PLAU, PTGER2, CHRM2, FKBP1A, TDP1, MAOA, TTR, XDH, NR1I2, ABCC1, ALOX12, EGLN1, RPS6KA3, PSMB1, PRKCZ, DUSP3, DRD2, SLC2A1, OPRM1, GPR17, F13A1, ROCK1, GRM5, CDK2, OPRD1, CAPN1, FFAR4, CHRNB4, DRD1, AAK1, DNMT1, PRKACA, RORB, LGALS3, SERPINE1, LYN, SCN9A, IL23R, KEAP1, STAT1, RPS6KA6, DUT, CETP, ACVRL1, GLO1, MAP2K2, CASP8, ZAP70, HDAC5, FCGRT, RET, PKN1, CHUK, FFAR2, ACACA, BRAF, STK3, SCD, ITGB1, ACE, FAAH, ERAP1, ABCB1, F2R, PTPN11, PIK3CB, ACVR1B, PIN1, GRB2, DRD3, HDAC11, YES1, KDM6B, ESR2, GRIA2, HIF1A, HPGD, GPBAR1, KDM4C, ANPEP, GUSB, NOS2, CXCR4, HSP90AA1, LDHB, PTPN7, METAP2, CFTR, NFE2L2, PDGFRB, CAMKK2, NPC1, GRK5, MME, HDAC2, CHKA, PDE3B, NT5E, DCUN1D1, NAAA, KDM4A, PDE11A, KIF11, CBX4, MIF, NOS3, HRH3, KCNA5, HTR2C, GABRA1, SCN4A, NLK, MC5R, AOC3, GPR35, ACHE, CHRM3, AKR1C1, RXFP1, ADRB1, P2RX4, TYRO3, CXCR6, S1PR4, TBXA2R, MDM4, ACACB, TACR1, TBK1, NOTUM, ENPP1, GBA, CCNE1, RAB9A, GLS, NOX1, GSTP1, PSMB2, MAOB, GBA2, CA3, PKN2, NQO2, TNIK, KLK5, FFAR1, RPS6KA5, TOP1, LNPEP, AKR1C2, FYN, CYP2A6, BRDT, TMPRSS6, KLK1, HTR7, PRSS1, MAP2K1, TAOK3, ULK1, TRPA1, PLK4, CCR1, CHEK1, ERN1, CCR2, PLA2G2A, EPHB2, SLC1A3, KLK7, FGR, KDR, PARP2, ADORA2A, DPP7, SLC1A1, CPT1B, SLC1A2, ADORA3, EPHX2, CASP6, FLT3, ITGB3, KIT, SOAT1, EPHB3, NR2E3, KCNK9, GRM4, CTSS, CHRNA1, MAP3K5, MAPK1, DOT1L, PIK3CD, P2RY12, ARG2, PTPN1, S1PR3, LTA4H, CCR5, CSNK2A2, CSNK2A1, CTSG, SLC40A1, F12, FAP, PARP1, OPRK1, ABL1, AKT2, TBXAS1, ST14, NAMPT, HTR2A, APLNR, CFD, MET, JAK2, PRKCA, IDE, AKT3, NR1H3, PRKCD, ADORA2B, HDAC8, SLC6A2, BMP2K, HDAC3, DYRK1B, PRKCQ, CDC7, PPIA, MAP3K11, FDPS, HDAC10, BCHE, CSK, CLK2, AXL, TYMS, HDAC9, ALOX5, HSD11B2, MC4R, F11, NTSR1, PREP, KCNH2, CDK7, CARM1, SLC6A4, PRMT6, MMP7, IKBKB, PAK4, TAOK1, HDAC4, ERAP2, NOS1, DYRK1A, EPHA4, ROCK2, DAO, ESR1, PKMYT1, HCAR2, BMPR1A, HSD11B1, GCK, MCHR1, P2RX7, NEK2, MAP4K4, IMPDH2, FLT1, ALK, CTSK, DCTPP1, HTR3A, CNR1, HDAC1, CDK4, F7, MAP3K14, CTSB, PIK3CA, FER, BCL6, UGT2B7, TEC, ICMT, IDO1, S1PR2, CTSL, PDPK1, P2RY2, TGM2, CD38, BCR, CAMK2D, PRMT1, PARG, ADAM17, PPARA, DPP4, IDH1, PIK3CG, RXRB, PRMT5, PRKAA1, VDR, MAP3K20, RIPK3, AHCY, SLC6A3, TDO2, TDP2, BRD4, ATAD2, SPHK2, EP300, F2, MARK3, GHSR, PDE7A, FABP4, RXRG, EGFR, TTK, ULK3, FKBP5, FBP1, GRM3, TGFBR2, MAPKAPK2, SLC5A1, SYK, PLK1, NMT1, APH1A, NTRK1, and MMP1.

Table S4 Non-volatile metabolites for yellowhorn treat enuresis, hyperlipidemia, neurodegenerative diseases, and prostatitis.

Source	Name	Classification	No.	Degree
Flower	Zarzissine	Alkaloids	Flower25	61
	2-Aminopurine	Nucleotides and derivatives	Flower31	53
	Hispidulin (5,7,4'-Trihydroxy-6-methoxyflavone)	Flavonoids	Flower11	51
	Luteolin-3'-O-glucoside	Flavonoids	Flower13	47
	Naringenin (5,7,4'-Trihydroxyflavanone)	Flavonoids	Flower4	47
	Diosmetin (5,7,3'-Trihydroxy-4'-methoxyflavone)	Flavonoids	Flower9	47
	Luteolin-7-O-gentiobioside	Flavonoids	Flower15	46
	3,5,4'-Trihydroxy-7-methoxyflavone (Rhamnocitrin)	Flavonoids	Flower16	46
	Isorhamnetin-3-O-rutinoside (Narcissin)	Flavonoids	Flower18	46
	Kaempferol-3-O-sambubioside	Flavonoids	Flower21	46
	Luteolin-4'-O-glucoside	Flavonoids	Flower14	45
	Kaempferol-3-O-galactoside (Trifolin)	Flavonoids	Flower19	45
	Kaempferol-3-O-glucoside (Astragalin)	Flavonoids	Flower20	45
	Diosmetin-7-O-glucoside	Flavonoids	Flower10	43
	6-C-MethylKaempferol-3-glucoside	Flavonoids	Flower12	43
	Rhamnetin-3-O-Rutinoside	Flavonoids	Flower24	43
	Kaempferol-7-O-glucoside	Flavonoids	Flower22	42
	Quercetin-3-O-rhamnoside(Quercitrin)	Flavonoids	Flower23	42
	Guanosine	Nucleotides and derivatives	Flower32	42
	L-Homocystine	Amino acids and derivatives	Flower30	41
	Vidarabine	Nucleotides and derivatives	Flower33	41
	Adenosine	Nucleotides and derivatives	Flower34	41
	Persicoside	Flavonoids	Flower5	41
	Isorhamnetin-3-O-neohesperidoside	Flavonoids	Flower17	40
	isoscopoletin	Lignans and Coumarins	Flower27	40
	Scopoletin (7-Hydroxy-6-methoxycoumarin)	Lignans and Coumarins	Flower28	38
	Gallic acid	Phenolic acids	Flower29	37
	Chrysoeriol-7-O-glucoside	Flavonoids	Flower8	37
	Savinin	Lignans and Coumarins	Flower26	36
	Hesperetin-7-O-glucoside	Flavonoids	Flower3	35
	Oleanolic acid-3-O-glucoside	Terpenoids	Flower7	35
	3,5,7-Trihydroxyflavanone (Pinobanksin)	Flavonoids	Flower6	34
	Shanzhiside methyl ester	Terpenoids	Flower1	32
	Oleanolic acid-3-O-glucoside	Terpenoids	Flower2	29
Leaf	Jujubogenin	Terpenoids	Leaf3	81
	Serotonin	Alkaloids	Leaf21	54
	Tryptamine	Alkaloids	Leaf19	48
	L-Lysine	Amino acids and derivatives	Leaf33	48
	Apigenin-7-O-neohesperidoside (Rhoifolin)	Flavonoids	Leaf10	47
	Epigallocatechin	Flavonoids	Leaf8	47

Table S4 Non-volatile metabolites for yellowhorn treat enuresis, hyperlipidemia, neurodegenerative diseases, and prostatitis (continued).

Source	Name	Classification	No.	Degree
Leaf	Gallocatechin	Flavonoids	Leaf9	47
	Apigenin-7-O-rutinoside (Isorhoifolin)	Flavonoids	Leaf11	46
	N-Methyltryptamine	Alkaloids	Leaf20	46
	Quercetin-3,7-Di-O-rhamnoside	Flavonoids	Leaf14	45
	DL-2-Aminoadipic acid	Alkaloids	Leaf18	44
	L-Valine	Amino acids and derivatives	Leaf31	44
	Myricetin-3-O-galactoside	Flavonoids	Leaf12	43
	Cannabiscitrin	Flavonoids	Leaf13	42
	L-Glutamine	Amino acids and derivatives	Leaf32	42
	Catechin	Flavonoids	Leaf6	42
	Epicatechin	Flavonoids	Leaf7	42
	N-Feruloylagmatine	Alkaloids	Leaf16	40
	Acetaminophen	Alkaloids	Leaf17	40
	Cis-Coutaric acid	Phenolic acids	Leaf26	38
	5-Glucosyloxy-2-Hydroxybenzoic acid methyl ester	Phenolic acids	Leaf28	38
	3-O-p-Coumaroylquinic acid	Phenolic acids	Leaf30	38
	Acetryptine	Alkaloids	Leaf22	36
	Isofraxetin	Lignans and Coumarins	Leaf24	36
	4-(3,4,5-Trihydroxybenzoxy)benzoic acid	Phenolic acids	Leaf29	36
	Hederagenin	Terpenoids	Leaf4	36
	Fraxetin-8-O-glucoside (Fraxin)	Lignans and Coumarins	Leaf23	34
	p-Coumaric acid-4-O-glucoside	Phenolic acids	Leaf25	33
	2-Nitrophenol	Phenolic acids	Leaf27	33
	Pomolic acid	Terpenoids	Leaf1	32
	30-Norhederagenin	Terpenoids	Leaf5	32
	2-Phenylethylamine	Alkaloids	Leaf15	31
	Corosolic acid	Terpenoids	Leaf2	29

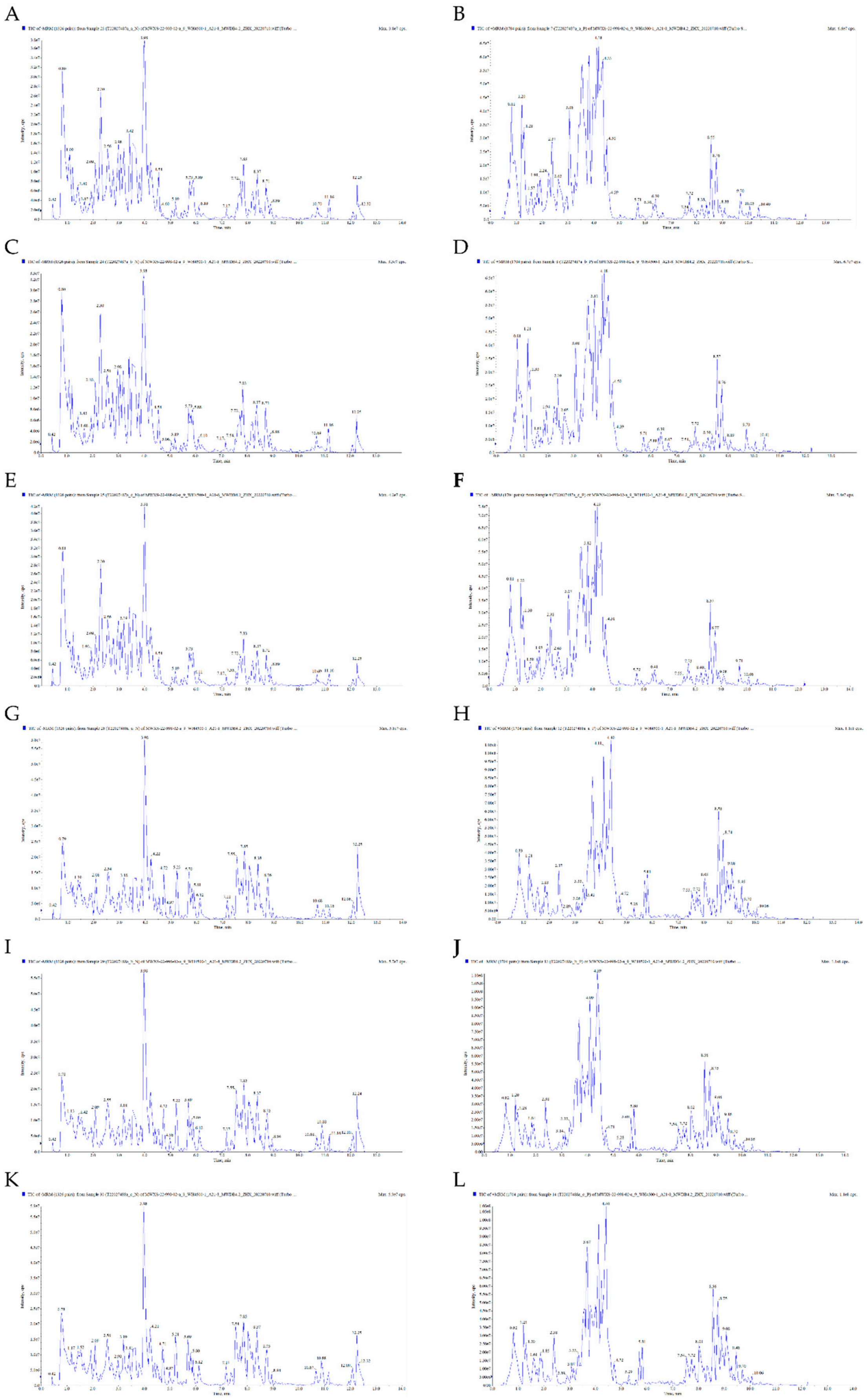
A
B
C
D
E
F
G
H
I
J
K
L

Figure S1 Total ions current of non-volatile metabolites in yellowhorn Leaf (A-F) and flower (G-L). (A, C, E, G, I, and K) Negative ion mode; (B, D, F, H, J, and L) positive ion mode.

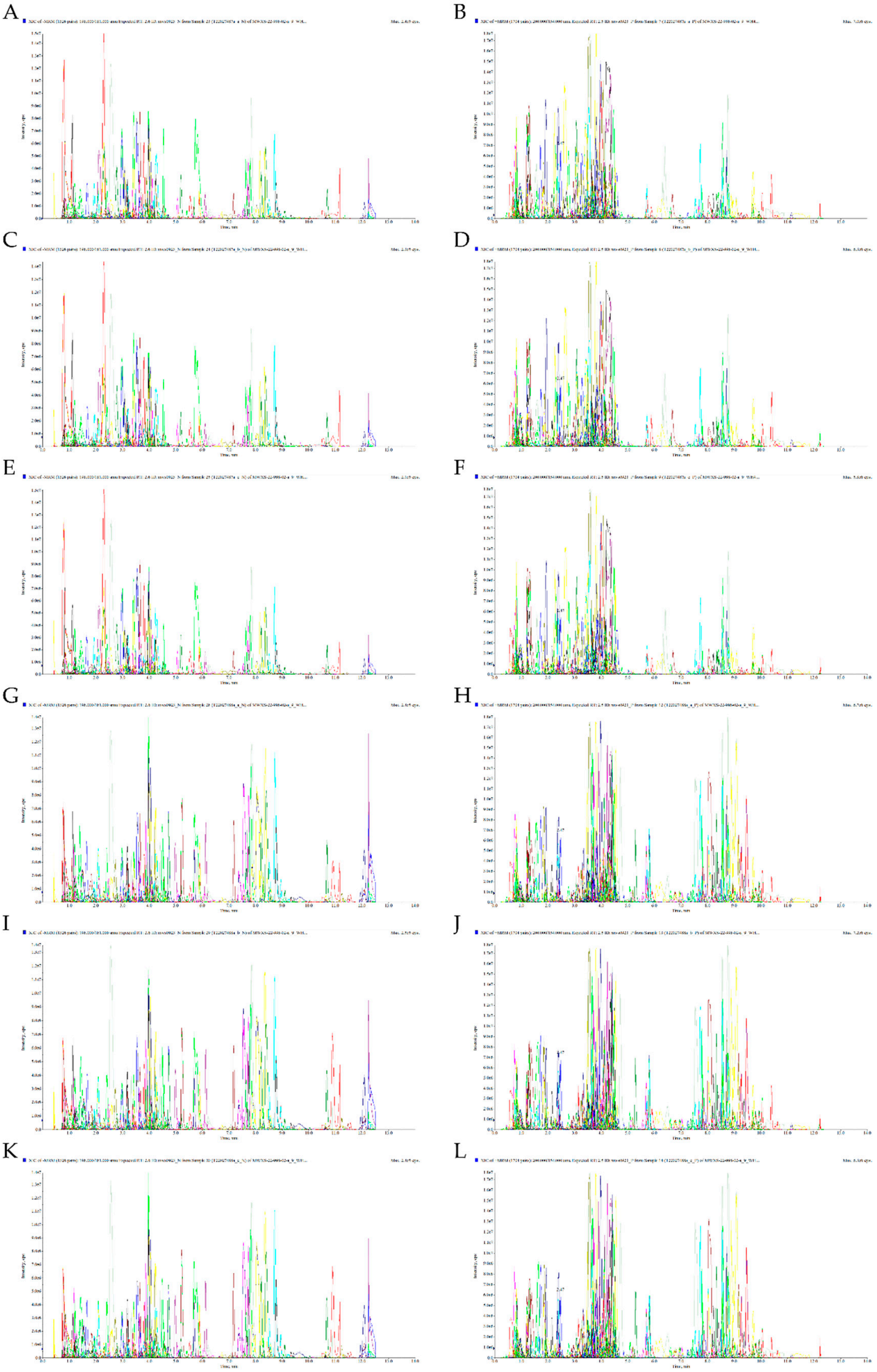

Figure S2. Multiple reaction monitoring non-volatile metabolite images

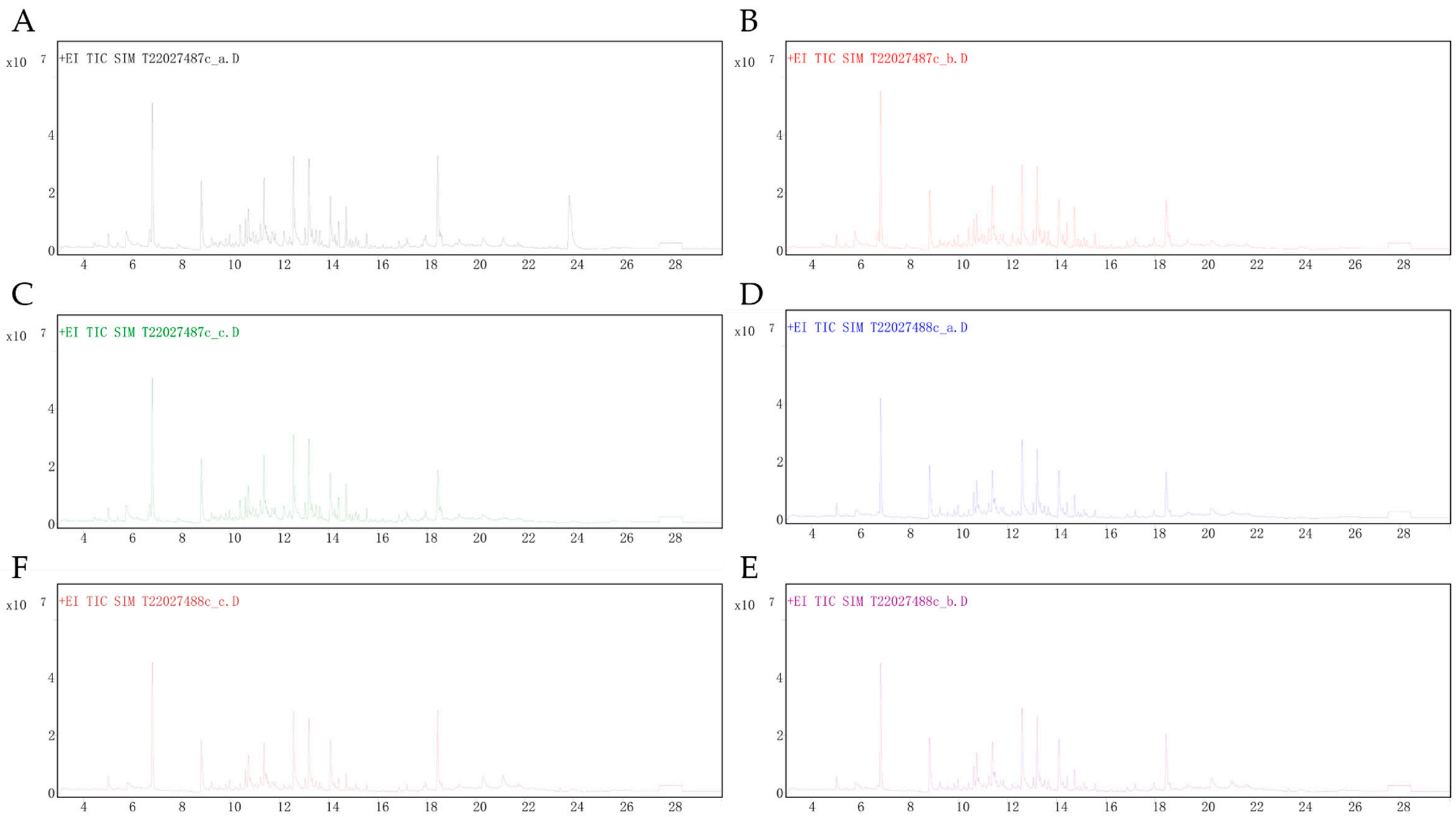

Figure S3. Total ions current of volatile metabolites in yellowhorn Leaf (A-B) and flower (D-E).

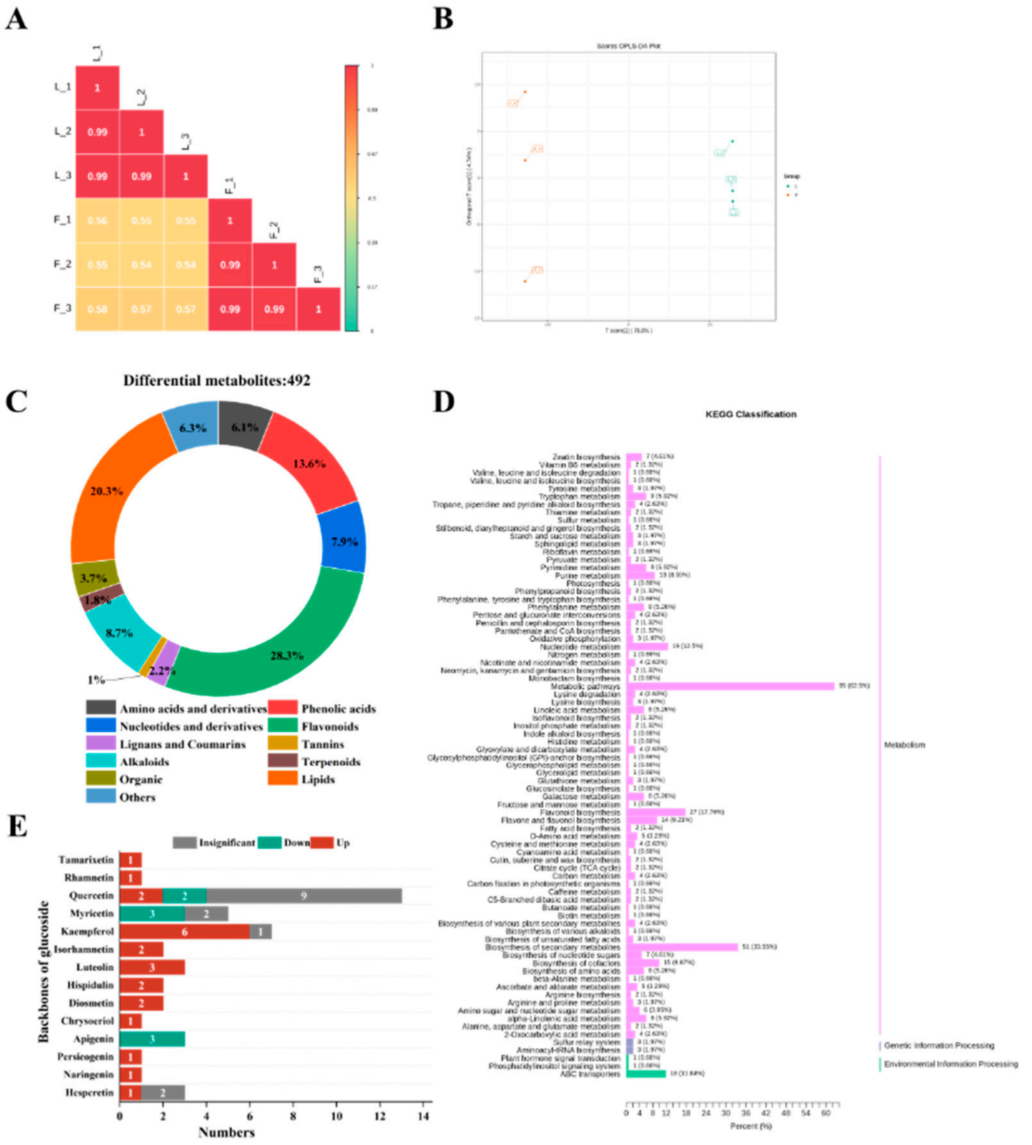

Figure. S4 Screening of significantly differentially expressed non-volatile metabolites in yellowhorn leaf and flower. (A) Correlation heat map of non-volatile metabolites of yellowhorn leaves and flowers; (B) Scores plots of OPLS-DA; (C) Proportion of different classes of differentially expressed non-volatile metabolites; (D) KEGG annotate ions and enrichment results of the differentially expressed non-volatile metabolites; (E) Differentially expressed non-volatile metabolites of yellowhorn leaves and flowers.
